# Intermolecular CH-π Electrons Interaction in Poly (9,9-dioctylfluorenyl-2,7-diyl) (PFO): An Experimental and Theoretical Study

**DOI:** 10.3390/molecules27051488

**Published:** 2022-02-23

**Authors:** Amin O. Elzupir, Rageh K. Hussein, Khalid H. Ibnaouf

**Affiliations:** 1 Deanship of Scientific Research, Imam Mohammad Ibn Saud Islamic University (IMSIU), Riyadh 13318, Saudi Arabia; aoalamalhuda@imamu.edu.sa; 2 Physics Department, College of Science, Imam Mohammad Ibn Saud Islamic University (IMSIU), Riyadh 13318, Saudi Arabia; rahussein@imamu.edu.sa

**Keywords:** conjugated polymers PFO, CH-π electrons interaction, hydrogen bonding, DFT, molecular doking model

## Abstract

This study demonstrates the presence of CH-π interaction in poly [9,9-dioctylfluorenyl-2,7-diyl] (PFO-1) due to an aggregate formation of PFO-1 in the liquid state. The absorption spectra of PFO-1 in certain solvents at low concentrations showed a single band at 390 nm. However, when using high concentrations, a new band at 437 nm appeared. This band is due to the aggregate formation of PFO-1. The aggregate formation occurs as a result of the CH interaction of the *n*-alkyl side chains with π-electrons in the benzene ring. The optical characteristics of another conjugated polymer of poly [9,9-di-(2-ethylhexyl)-fluorenyl-2,7-diyl] (PFO-2) were investigated to confirm the CH-π interaction. The absorption showed only one wavelength at 390 nm without any new band at the end of the spectrum, even at higher concentrations and lower temperatures. The main reason for the absence of aggregate formation in PFO-2 is the sterical hindrance caused by the branched alkyl side chains. In addition, Density Functional Theory (DFT) was used to compute the HOMO–LUMO transitions, electron charge distribution, and frontier molecular orbitals for each polymer. The Mulliken charge distribution and demonstrated a notable difference in the reactivity of the alkyl side chain, confirming the higher ability of PFO-1 to form CH-π bonds. docking model emphasized that the band at 437 nm could be attributed to the interaction between CH in the *n*-alkyl side chain and π bonds in the aromatic rings of PFO-1.

## 1. Introduction

Recently, conjugated polymers have become essential for researchers and scientists because of their high activity as materials in different disciplines in electronic and optoelectronic devices [1,2,3]. Polymers containing polyfluorene functionality have attracted the most attention, because they exhibit strong absorption and fluorescence emission cross-sections, high fluorescence quantum yields, and good photochemical stability. Furthermore, what distinguishes this polymer is that it has broad spectra suitable for tunable lasers [4,5,6,7,8,9,10]. The optical properties of polyfluorene polymers, in general, are strongly affected by the nature of the solvent, temperatures, and structural properties of the conjugation moiety [11,12,13].

**Figure 1:**
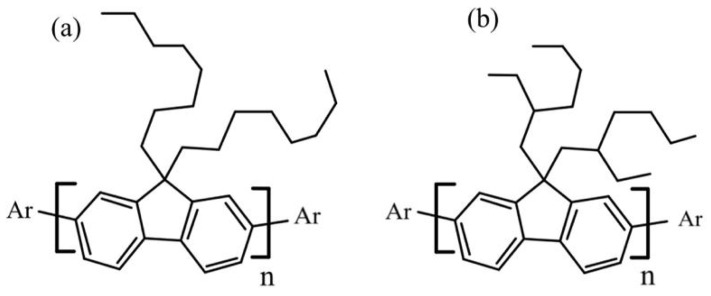
Chemical structure of (**a**) PFO-1 and (**b**) PFO-2.

Researchers have become interested in studying the spectral properties of some conjugated polymers, particularly the promising conjugated polymers of poly [9,9-dioctylfluorenyl-2,7-diyl] (PFO-1) and poly [9,9-di-(2-ethylhexyl)-fluorenyl-2,7-diyl] (PFO-2) (Figure 1). The main distinguishing feature of PFO-1 is that it contains an *n*-alkyl side chain, while PFO-2 has a branched alkyl side chain. The literature discusses their structural features deeply in the ground and excited states, such as the effect of the molecular weight of PFO on the structure of a single chain and the formation of α and β phase conformations. The conformer β phase is known to be formed due to the crystallization of the *n*-alkyl side chains, and has a more significant intrachain torsion angle of 165° than that of α phase 135° [14,15,16,17,18,19]. Despite a few unresolved issues, such as the bond types for dimers and/or aggregates, there is still a lack of fundamental functional groups involved in such a reaction. In addition, some other PFO derivatives, such as PFO-2, could not produce dimers and/or aggregates. To shed light on these questions, PFO-1 and PFO-2 were studied in their ground and excited states, paying particular attention to the bonds responsible for the aggregation attributed to the β phase conformation. This work demonstrates evidence of the presence of the CH-π interaction and the confirmation analysis of polymer PFO-1 in the ground state. Furthermore, the influence of the alkyl group attached to PFO was studied in terms of the presence (PFO-2) or absence (PFO-1) of the steric hindrance. Furthermore, the energy band gaps, molecular orbitals, HOMO–LUMO interaction, and charge distribution of these polymers were calculated. A molecular docking model indicates that the band at 437 nm is caused by an interaction between CH-π bonds of PFO-1.

## 2. Experimental Section

### 2.1. Steady State

The polymers (PFO-1) and (PFO-2) were purchased from American Dye Source. Their molecular weights were in the ranges 40,000–150,000 and 10,000–75,000, respectively. Their molecular structures are given in Figure 1. The absorption and fluorescence spectra were studied using different concentrations and temperatures. A Perkin Elmer Lambda 40 spectrophotometer was used at 200 to 800 nm to determine the absorption spectra. The fluorescence spectra were monitored by a Perkin Elmer LS45 spectrofluorometer (200–900 nm).

### 2.2. Computational Method

Density Functional Theory (DFT) was used to determine the equilibrium structure using ab initio levels to support these results. The calculations were performed using the Gaussian 09 package [20]. Minimum energy-optimized structures for each PFO were obtained by ab initio density functional theory (DFT). The B3LYP method alongside 6-31G** basis sets was used to estimate HOMO–LUMO transitions, frontier molecular orbitals, dipole moment, electron charges distribution [21]. For the molecular docking model, the Auto Dock Vina plugin UCSF Chimera was used.

## 3. Result and Discussion

**Figure 2:**
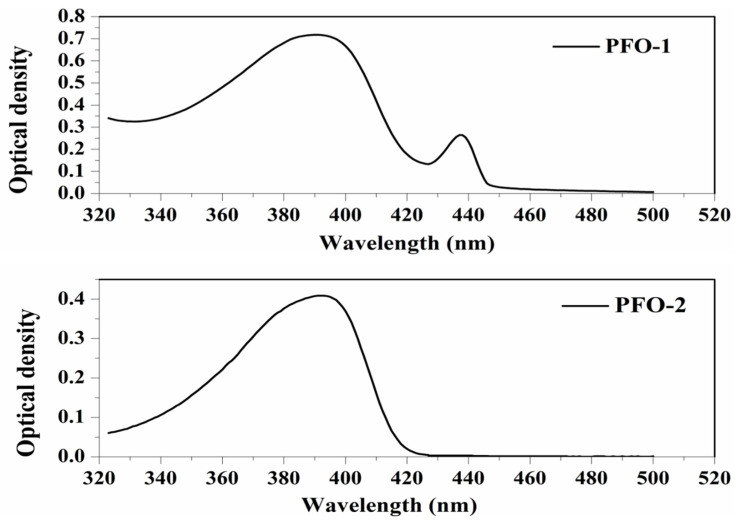
Absorption spectrum of PFO-1 and PFO-2 in tetrahydrofuran.

**Figure 3:**
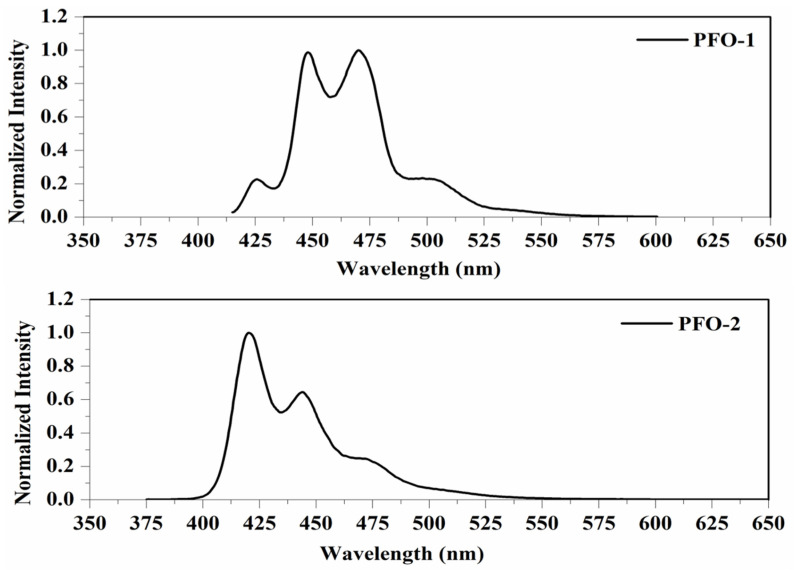
Fluorescence spectrum of PFO-1 and PFO-2 in tetrahydrofuran.

PFO-1, under lower concentrations, showed broadband absorption around 390 nm. This band comprises two peaks: one at 385 nm and another at 410 nm. PFO-1 showed a new absorption band at higher concentrations under the same operational conditions at 437 nm, as shown in Figure 2. It is worth noting that the optical density of this band increases with increasing concentration. Furthermore, the effect of the temperature on PFO-1 showed that the optical density of the band 437 nm decreases significantly with increasing temperature (See Figure S1). All of these experiments provided strong evidence that the band at 437 nm was due to aggregate formation. The emission spectra of PFO-1 showed three bands at 422, 445, and 470 nm, as shown in Figure 3. These bands could be ascribed to the absorption bands at 385, 410, and 437 nm, respectively. When the concentration increased, the emission spectrum was drastically changed, where the band 470 nm grew faster and became dominant. These results are consistent with what has previously been reported. Some researchers have attributed the appearance of bands 390 and 437 nm in the absorption spectrum to the α and β phase conformations, respectively [14]. 

On the other hand, PFO-2 showed an absorption band at 390 nm, and there was no appearance of the band 437 nm even at high concentrations and low temperatures, as seen in Figure 2. The emission spectra of PFO-2 exhibited two distinct bands at 422 and 445 nm, as illustrated in Figure 3. These bands may be due to the absorption bands at 385 and 410 nm, respectively. It can be seen that at the end of the spectrum, there was a tiny band at 470 nm, which indicates that aggregates were formed in a minimal quantity, which is less than the limit of detection of the UV-Vis spectrophotometer.

Recent studies have shown that carbon-linked hydrogen (CH) can form hydrogen bonds with π-electrons in the benzene ring and other related heterocycles [22,23,24,25,26]. It is well known that the pKa for *n*-alkyl side chains attached to PFO is about 50 [27,28]. Therefore, it is possible to form hydrogen bonds with π-electrons in the benzene rings due to the high conjugation of PFO, which makes the π-electrons more accessible to protons in the *n*-alkyl side chains. However, the molecular structure of the PFO and its derivatives lacks the fundamental functional groups involved in aggregate formation. Therefore, the hydrogen bonds cannot be formed for the low molecular weights, such as PFO or PFO monomers [14,29,30]. These results are consistent with those previously reported. The absence of the formation in monomer molecules may be due to the lack of electrons available in the electronic cloud, which increases with increasing molecular weight. On the other hand, the aggregate formation was not observed in PFO-2, because it contains two-branched alkyl groups that lead to steric hindrance, which prevents the formation of hydrogen bonds.

### Theoretical Calculations

**Figure 4:**
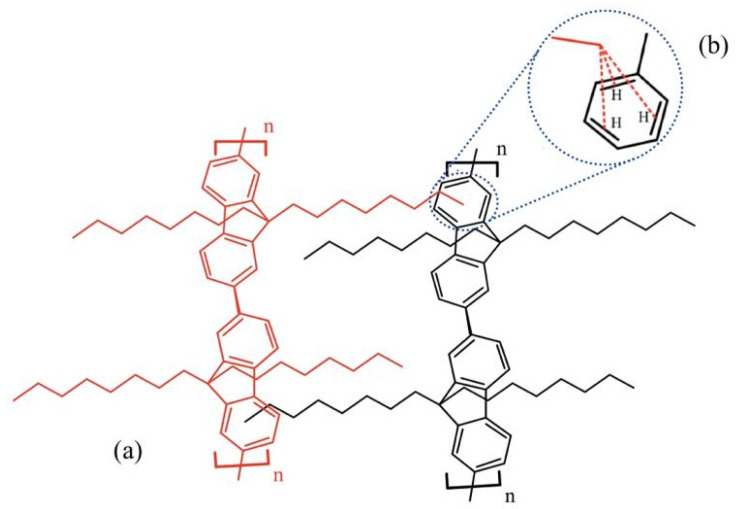
(**a**) Possible CH-π electrons interactions in a structure of PFO-1. (**b**) CH-π electrons interaction.

The experimental results were confirmed by computational methods based on DFT. PFO-1 with an *n*-alkyl side chain (as two monomers) and PFO-2 with a branched alkyl side chain (as two monomers) were studied. The results showed that the *n*-alkyl side chain of the PFO-1 was straight and perpendicular to the funeral ring. In contrast, the branched alkyl chain in PFO-2 wraps around the polymer, preventing the interaction between the alkyl group and the π-system with another PFO-2 molecule. The calculated central plane angles between the two monomers in each PFO are presented in Table 1. The central plane angle of PFO-1 is greater than that of PFO-2. That means the electron clouds will be more easily delocalized in PFO-1, and the electrons will become more accessible for attachment to the protons in the *n*-alkyl side chain. The possible interactions of the CH-π electrons in PFO-1 was presented in Figure 4.

**Figure 5:**
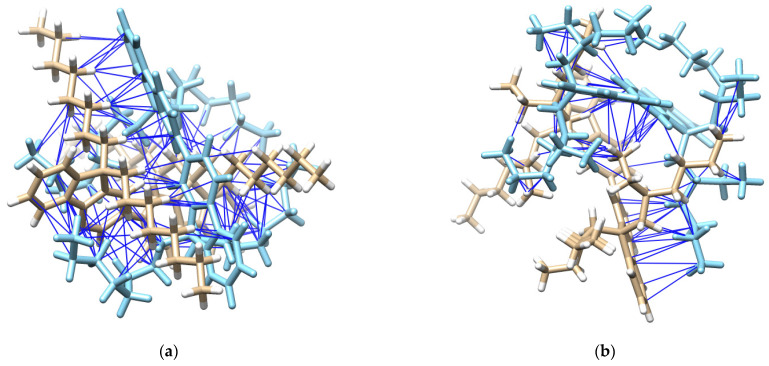
(**a**) Possible interactions in PFO-1 docked with an optimized structure of PFO-1, and (**b**) possible interactions in PFO-2 docked with an optimized structure of PFO-2.

Furthermore, PFO-1 and PFO-2 were docked—with themselves—using Auto Dock Vina plugin UCSF Chimera [31,32]. PFO-1 showed good interaction bonds, resulting from *n*-alkyl side chain interactions with the aromatic sides, with a very low binding affinity score of −9.1 kcal/mol (See Figure 5). This result supports our assumption that aggregate formation is a result of the interaction between CH in an *n*-alkyl side chain and π bonds in the aromatic rings. In contrast, PFO-2 showed fewer interaction modes between the branched alkyl side chain and the aromatic sides with the binding affinity score of −6.1 kcal/mol. The van der Waals interactions are depicted with blue lines in Figure 5. Moreover, most of these interactions are due to the branched alkyl side itself with another alkyl side in PFO-2. These findings confirm our hypothesis on the influence of strike hindrance prevents PFO-2 from forming in an aggregate formation. 

**Figure 6:**
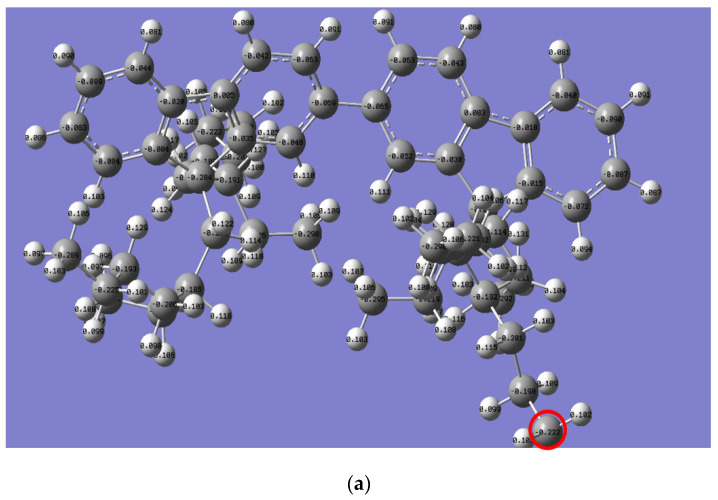

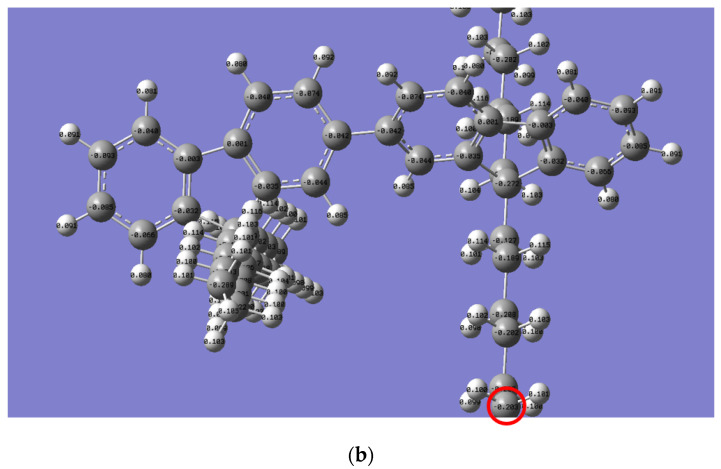
Mulliken charge is for the electrons distribution of (**a**) PFO-1 and (**b**) PFO-2.

The Mulliken charge distribution revealed a significant difference in the reactivity of the alkyl side chain for both polymers, as presented in Figure 6. Interestingly, the electron charge of the last carbon in the *n*-alkyl side chain for PFO-1 is lower than that of PFO-2 (highlighted by a red circle). Thus, their electrophilicity is higher than the corresponding PFO-2. Consequently, PFO-1 has a greater ability to form CH-π bonds.

**Figure 7:**
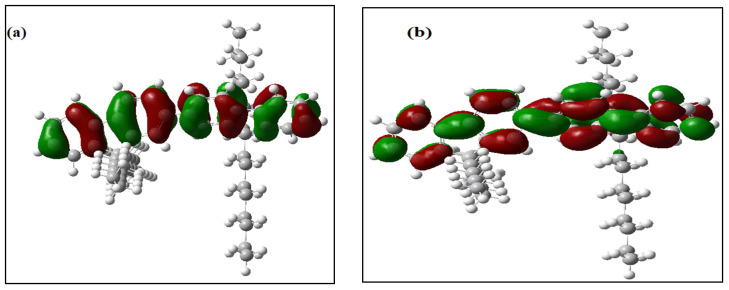

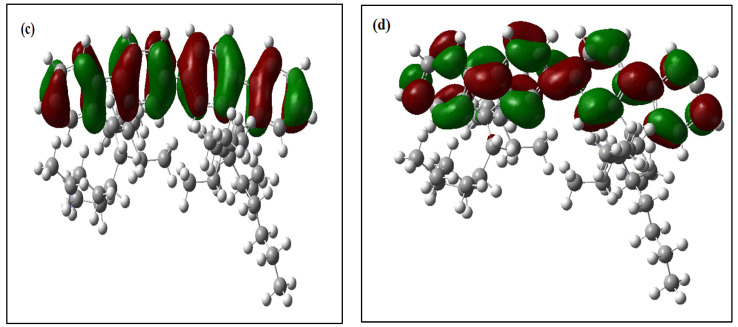
Frontier molecular orbitals of (**a**) HOMO of PFO-1, (**b**) LUMO of PFO-1, (**c**) HOMO of PFO-2 and (**d**) LUMO of PFO-2.

**Figure 8:**
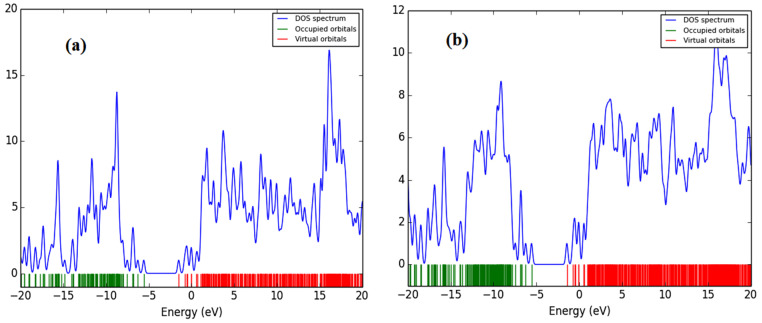
The energy band gap of (**a**) PFO-1 and (**b**) PFO-2.

All of the theoretical data, including HOMO and LUMO transitions, total energy (E_t_), and energy band gaps (E_g_), are listed in Table 1. The depreciation of the Et of PFO-1 means it is more susceptible to aggregate formation. In addition, the HOMO–LUMO interactions of these polymers are illustrated in Figure 7. The HOMO–LUMO interactions and the decrease in Eg are strong indications that PFO-1 is more reactive, and therefore more capable of forming hydrogen bonds, as shown in Figure 8. 

## 4. Conclusions

In conclusion, we explained for the first time the possibility of forming hydrogen bonds between *n*-alkyl groups and the benzene rings in PFO-1, implementing experimental and theoretical approaches. At lower concentrations, PFO-1 showed an absorption band at 390 nm. In addition, a new band at 437 nm was observed that increased with higher concentrations. Moreover, the impact of temperature on this formed band was noticed and was found to be inversely proportional to it. In line with our expectations, these findings demonstrated the aggregate formation of PFO-1. To further support our claim, density functional theory and docking models were used. The obtained results emphasized that the band at 437 nm could be attributed to the interaction between CH in the *n*-alkyl side chain and π bonds in the aromatic rings of PFO-1. Taken together, we envision that our results may shed light on many other physical and chemical behaviors related to these or other polymers. 

## Figures and Tables

**Table 1:** Theoretical calculations of the total energy E_t_, and the energy of HOMO and LUMO for two monomers of PFO.

	PFO-1	PFO-2
E_T_ (eV)	−61,461.607	−61,502.042
E_HOMO_	−5.484	−5.564
E_LUMO_	−1.427	−1.446
ΔE_g_	4.057	4.118
Central plane angle	142.088	139.328

## Data Availability

The data presented in this study are available in the supplementary material.

## Supplementary Materials

The following supporting information can be downloaded online. Figure S1: Absorption spectra of PFO-1 as a function (A) Temperature (B) Concentration.
