# Supplementary material for: Intermolecular CH-π Electrons Interaction in Poly (9,9-dioctylfluorenyl-2,7-diyl) (PFO): An Experimental and Theoretical Study
Source: Molecules. 2022 Feb 23;27(5):1488. doi: 10.3390/molecules27051488 (PMC8912045; doi:10.3390/molecules27051488)
Supplement: Supplementary file 1 [file molecules-27-01488-s001.zip › molecules-1605899-supplementary.pdf]

Article

Intermolecular CH-π Electrons Interaction in Poly (9,9-dioctylfluorenyl-2, 7-diyl) (PFO): An Experimental and Theoretical Study

Amin O. Elzupir [1], Rageh K. Hussein [2] and Khalid H. Ibnaouf [2,*]

[1] Deanship of Scientific Research, Imam Mohammad Ibn Saud Islamic University (IMSIU), Riyadh 13318, Saudi Arabia; aminosman81@gmail.com

[2] Physics Department, College of Science, Imam Mohammad Ibn Saud Islamic University (IMSIU), Riyadh 13318, Saudi Arabia; rahussein@imamu.edu.sa

* Correspondence: khiahmed@imamu.edu.sa

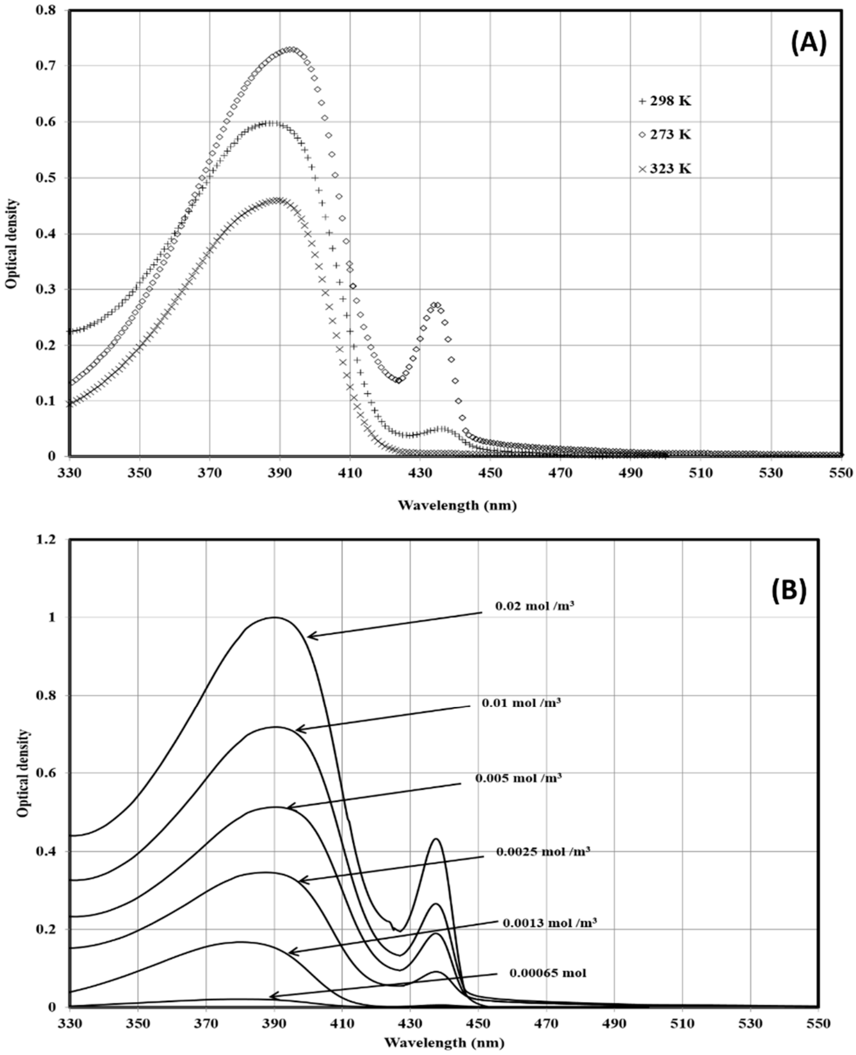

Figure S1: Absorption spectra of PFO-1 as a function (A) Temperature (B) Concentration
